# Integrated transcriptome and metabolome analysis unveils the mechanism of color transition in *Camellia reticulata* ‘Tongzimian’

**DOI:** 10.3389/fpls.2026.1831409

**Published:** 2026-04-23

**Authors:** Lin Zhou, Wen-kun Su, Zi-yan Liu, Shi Liang, Zhi Ou, Yan Qu

**Affiliations:** College of Landscape Architecture and Horticulture Science, Yunnan Province Engineering Research Center for Functional Flower Resources and Industrialization, Yunnan Key Laboratory of Landscape Plant Resource Cultivation and Application, Southwest Forestry University, Kunming, China

**Keywords:** Camellia reticulata, color fading, CrANS, metabolome, transcriptome

## Abstract

*Camellia reticulata* ‘Tongzimian’ is the lightest-colored cultivar within the species, yet the molecular mechanism underlying its color formation remains elusive. In this study, ‘Tongzimian’ was used to observe phenotypic characteristics during flowering. Through multi-omics analyses, key substances and genes associated with petal fading were identified, clarifying the role of the *CrANS* gene in regulating floral coloration. Targeted anthocyanin metabolomics showed that procyanidin is the main pigment in ‘Tongzimian’ petals, with cyanidin serving as the key pigment driving color transformation. Transcriptome analysis revealed that low CrANS expression at full bloom is the primary factor reducing cyanidin content. Overexpressing *CrANS* demonstrated that the gene strongly promotes anthocyanin synthesis in tobacco corollas, thereby deepening flower color. The study suggests that a balance between cyanidin and procyanidin is central in regulating petal color transformation. Collectively, this research provides a novel framework for flower color dilution and supports molecular breeding of flower color in *C. reticulata*.

## 1 Introduction

*Camellia reticulata* is a traditional woody plant found in China (Qiu et al., 2014; Xu et al., 2024). It is renowned for its beautiful shape and colorful flowers (Tong and Gao, 2020), earning it the title of “The champion flower in the clouds.” Among its cultivars, *C. reticulata* ‘Tongzimian’ stands out as one of the rarest and lightest-colored (Qu et al., 2024). When it first flowers, it is pale pink with a reddish tinge, gradually turning white after it fully blooms (Xiao et al., 2025). This color transformation adds considerable ornamental value to ‘Tongzimian’. While some studies have investigated the flower colors of ‘Tongzimian’ (Geng et al., 2022), research on the molecular mechanisms behind their formation remains limited. Therefore, this study focuses on elucidating the factors and molecular mechanisms influencing floral color in this species.

The development of floral color is influenced by petal tissue structure (Abudayeh et al., 2022), vacuole pH (Chen et al., 2024), pigment type and content in petal cells (Zan et al., 2024), and environmental factors, with pigment accumulation playing a dominant role. Notably, previous studies report that cyanidin-type anthocyanins (Li et al., 2013, 2014), especially cyanidin-3,5-O-diglucoside (Wang et al., 2022), dominate in *Camellia* species and are the main pigments responsible for red floral color. The synthesis and accumulation of anthocyanins and their precursors are catalyzed by enzymes and proteins encoded by various structural genes, which directly determine both the type and quantity of anthocyanins present. For example, the bicolor phenotype of *Dahlia variabilis* ‘Yuino’ is caused by post-transcriptional silencing of the *DvCHS2* gene in white petal sectors, thereby blocking the anthocyanin biosynthetic pathway (Ohno et al., 2017, 2011). Similarly, in *Paeonia suffruticosa* cultivars ‘Qing Hai Hu Yin Bo’ and ‘High Noon’, petal pigmentation is closely linked to the spatiotemporal expression of *PsCHS* and *PsANS*, respectively, both of which influence anthocyanin accumulation (Gu et al., 2019; Luan et al., 2022). Furthermore, in the *Nelumbo nucifera* cultivar ‘Da Sajin’, degradation of the NnUFGTs protein in white petal regions prevents normal anthocyanin accumulation, resulting in a bicolor pattern (Deng et al., 2023).

Flower color variation describes changes in petal color across different stages of a plant’s flowering, endowing the plant with dynamic beauty. While the molecular mechanisms underlying petal color variation are being gradually unraveled, it is important to note that these mechanisms vary across plant species. For example, during flowering, the petals of *Lonicera japonica* (Pu et al., 2020) and *Edgeworthia chrysantha* (Zhou et al., 2023) shift from white to yellow, a transformation closely linked to changes in carotenoid content. In another case, *Paeonia suffruticosa* ‘Lv Mu Yin Yu’ exhibits petals shifting from green to white or pink as flowering progresses, a process tightly linked to the metabolic balance between chlorophyll degradation and anthocyanin synthesis (Hao et al., 2025). Conversely, *Syringa oblata* displays petal color fading due to reduced *SoUFGT2* transcription levels, which in turn reduce anthocyanin biosynthesis and lead to petal discoloration (Wang et al., 2025).

In this study, we investigated the physiological mechanisms underlying color changes by analyzing phenotypic characteristics and intracellular physiological changes at different coloration stages (the full coloring and full-bloom stages). Meanwhile, we combined transcriptomic and metabolomic analyses to explore the mechanism of ‘Tongzimian’ color formation and to determine the function of the key gene *CrANS*. These results provided a theoretical basis for breeding *C. reticulat*a cultivars of different colors. They also serve as a theoretical reference for studying the fading mechanism of flowers of other ornamental plants.

## 2 Materials and methods

### 2.1 Plant materials

Petals of *C. reticulata* ‘Tongzimian’ were obtained from the Nursery Base of Southwest Forestry University in Kunming, Yunnan Province, China (102°45’53”E, 25°4’0”N). From early January to late February 2022, the petals at different stages of opening (P1: full coloring stage; P2: full bloom stage) were collected from the ‘Tongzimian’ cultivar. In addition, transgenic and wild-type (WT) tobacco lines were cultivated at the same location, and corollas were collected at the full-bloom stage. All samples mentioned above were frozen immediately in liquid nitrogen and then stored at -80 °C for further use.

### 2.2 Flower color phenotyping and electron microscope scanning

After collecting the petals from the P1 and P2 stages of the ‘Tongzimian’, the color of the middle part of the complete petals in the middle layer was compared using RHSCC, with 10 replicates. The most frequently observed result was recorded as the final flower color. To further quantify color characteristics, the lightness (L*), red-greenness (a*), and yellow-blueness (b*) values of these petal samples were determined using a colorimeter (RM200QC, X-rite Incorporated), with three technical replicates per flower and three biological replicates per stage. Fresh petals of ‘Tongzimian’ at the P1 and P2 stages were placed in electron microscopy fixative, pressed with cotton, and sent to Wuhan Saiweier Biotechnology Co. for scanning electron microscopy.

### 2.3 Determination of the intracellular environment

Fresh medial petals weighing 5 g were cut into pieces and placed into a mortar and pestle. Silica was added, and the petals were ground into a homogeneous paste. The pH of the paste was determined using a pH meter (a-AB23PH ZH, OHAUS Instruments Changzhou Co.). Soluble protein and soluble sugar contents were measured in medial petals at P1 and P2, with three biological replicates per stage, using the Thomas Blue Protein Content Assay Kit and the Plant Soluble Sugar Content Kit (Suzhou Keming Biotechnology Co).

### 2.4 Targeted metabolome testing and analysis

Petals from the full coloring and full bloom stages of ‘Tongzimian’ and the full bloom stage of ‘Shizitou’ were sent to Wuhan Meitville Biotechnology Co., Ltd. for anthocyanin-targeted metabolome assay, with three biological replicates for each sample, which were detected by liquid chromatograph mass spectrometry (LC-MS/MS) technique.

To analyze anthocyanins, fresh petal samples were first freeze-dried and then ground to powder form (30 Hz, 1.5 min) using a ball mill. Next, 50 mg of the powder was weighed and dissolved in 500 μL of extract (50% aqueous methanol containing 0.1% hydrochloric acid), followed by vortexing for 5min, ultrasonication for 3 min, and centrifugation for 3min at 4 °C and 12000 r min^-1^. The supernatant was subsequently aspirated; this extraction step was repeated once, then the combined supernatants were filtered through a 0.22 μm membrane and stored in a feed bottle. For data acquisition, Ultra-performance liquid chromatography (UPLC) and Tandem Mass Spectrometry (MS/MS) were used: column, ACQUITY BEH C18; flow rate, 0.35 mL/min; injection volume, 2 μL. A 0.1% aqueous formic acid solution (A) and a 0.1% methanol-formic acid solution (B) served as mobile phases. The phase B proportion started at 5% at 0 min, increased to 50% at 6.00 min, to 95% at 12.00 min, held for 2 min, and was then decreased to 5% at 14 min, followed by 2 min for equilibration. For mass spectrometry, Electrospray Ionization (ESI) at 550 °C and Mass Spectrometry Voltage (MSV) at 5500 V in Positive Ion Mode (PIM) were applied, with Curtain Gas (CUR) at 35 psi. In Q-Trap6500+, each ion pair was scanned and detected according to the Declustering Potential (DP) and Collision Energy (CE).

Both qualitative and quantitative anthocyanins were analyzed against standards to build a Metware Database (MWDB). Mass spectrometry data were processed using Analyst 1.6.3 and MultiQuant 3.0.3 software: raw data were first qualitatively analyzed and then quantitatively processed. Retention times and peak profiles of reference standards were used to correct analyte peaks by integration, ensuring accurate qualitative and quantitative analysis. Standard solutions were prepared at concentrations from 0.01 ng/mL to 5000 ng/mL, and chromatographic peak intensity data were collected for each. Standard curves were plotted with concentration on the x-axis and peak area on the y-axis. Integrated peak areas of all detected samples were substituted into the standard curve equation for calculation. The final content of substances in actual samples was determined using the formula: Content (μg/g) = c*V/1000000/m.

### 2.5 Transcriptome analysis

Transcriptome sequencing was performed at Wuhan Metavir Biotechnology Co. First, RNA was extracted, and then libraries were prepared. Sequencing was then performed as described by Qu et al (Qu et al., 2024). RNA-seq sequencing was performed on the Illumina platform; each sample yielded approximately 6.5 Gb of valid data, and the Q30 score for all samples was ≥92% (**Supplementary Table 1**). Subsequently, the full-length de-redundant transcripts were used as reference sequences, and the clean reads from each second-generation sequencing sample were aligned to them.

All qualified transcripts were annotated against functional databases, including NR, SwissProt, KEGG, and GO. Transcription or gene expression levels were determined using FPKM (fragments per kilobase of transcript per million mapped reads). Differentially expressed genes (DEGs) were identified using the following filter conditions: |log_2_(FC)| ≥ 1 (fold change) and a false discovery rate (FDR) *p-value* < 0.05.

### 2.6 *CrANS* gene analysis

The CrANS homolog protein sequences from other plant species were obtained from the NCBI database. A phylogenetic tree was constructed in MEGA (MEGA 11) using the neighbor-joining (NJ) method with 1000 bootstrap replicates. Sequence alignment was performed using GeneDoc (GeneDoc 2.7).

### 2.7 *CrANS* cloning, vector construction, subcellular localization

The full-length sequences of *CrANS* were cloned from the cDNA of *C. reticulata* ‘Tongzimian’. The primers used were CrANS-F (ATGGTGGCTACTGTGGCA) and CrANS-R (TCATTTCCCTCCAAGCACC). PCR amplification was conducted by Green Tap Mix (Vazyme, Nanjing, China). After amplification, the correct bands were recovered from agarose gel electrophoresis and ligated into the pMD19-T vector (Takara, Beijing, China) for sequencing. Subsequently, the alignment sample sequence was compared with the full-length transcriptome of *C. reticulata* to confirm consistency before subsequent experiments.

To construct the pBWA(V)HS-CrANS-Glosgfp vector, Wuhan Boyuan Biotechnology Co., Ltd. first digested the vector with BsaI and then purified it using a PCR purification kit. Next, the purified vector was ligated with the PCR product. For transformation, 10 µL of the ligation product was introduced into DH5α E. coli competent cells. The transformed cells were then evenly spread onto Petri dishes containing Kan resistance and cultured at 37 °C. After 12 hours, 10 plaques were simultaneously picked for PCR identification. Following PCR, the bacterial liquids corresponding to bands 1–3 were selected. Of these 100 µL was sent for sequencing, while the remaining 400 µL was inoculated into LB medium containing 10 mL of kanamycin (50μg/mL) and cultured by shaking. Once sequencing results were available, the correct sequence was selected, and the plasmid was extracted.

After plasmid extraction, the pBWA(V)HS-CrANS-Glosgfp and Marker plasmids were injected into the lower epidermis of tobacco leaves. Then, the plants were incubated in low light for two days. Following incubation, the leaves were prepared as slides and examined using a laser confocal microscope (Nikon, Tokyo, Japan).

### 2.8 Stable tobacco transformation

The recombinant plasmid pBWA(V)HS-CrANS-GlosgGFP was introduced into GV3101 via electroporation. Leaf discs of the Nicotiana tabacum ‘K326’ tobacco cultivar were transformed using the GV3101-mediated method according to the Huazhong Agricultural University protocol (Ning et al., 2012). Hygromycin (25 mg/L), an antibiotic, was added to the MS medium to screen for transgenic lines. Plants overexpressing *CrANS* showing obvious color changes in their flowers were selected for further analysis.

### 2.9 Quantitative real-time polymerase chain reaction analysis

Total RNA was extracted using the Omega Plant RNA Kit (Omega, Massachusetts, America), following the manufacturer’s instructions. First-strand cDNA was synthesized using 5 All-In-One RT MasterMix (abm, Shanghai, China) according to the manufacturer’s protocol. The qRT-PCR reactions were performed using the Light Cycler 480 II. The 2^-ΔΔCt^ method was used, with *CrActin* and *NtActin* as the internal standards. The qRT-PCR primer sequences are shown in **Supplementary Table 2**.

### 2.10 Determination of anthocyanins in tobacco corollas

To determine anthocyanin in tobacco corollas with a Plant Anthocyanin Assay Kit (Suzhou Keming Biotechnology Co), weigh 0.1 g of corollas. Add 1 mL of extraction solution, homogenize, and transfer the entire mixture to an EP tube. Adjust volume to 1 mL, seal, and incubate at 75 °C for 2 minutes. Centrifuge at 8000 rpm for 10 minutes at room temperature and collect the supernatant for analysis.

### 2.11 Statistics analysis

In this study, triplicate data collection and t-tests revealed statistically significant differences between the experimental and control groups. Statistical significance is indicated: *P < 0.05, **P < 0.01, ***P < 0.001, ****P < 0.0001.

## 3 Results

### 3.1 The phenotypic characteristics and intracellular environment of ‘Tongzimian’ petals

**Figure 1:**
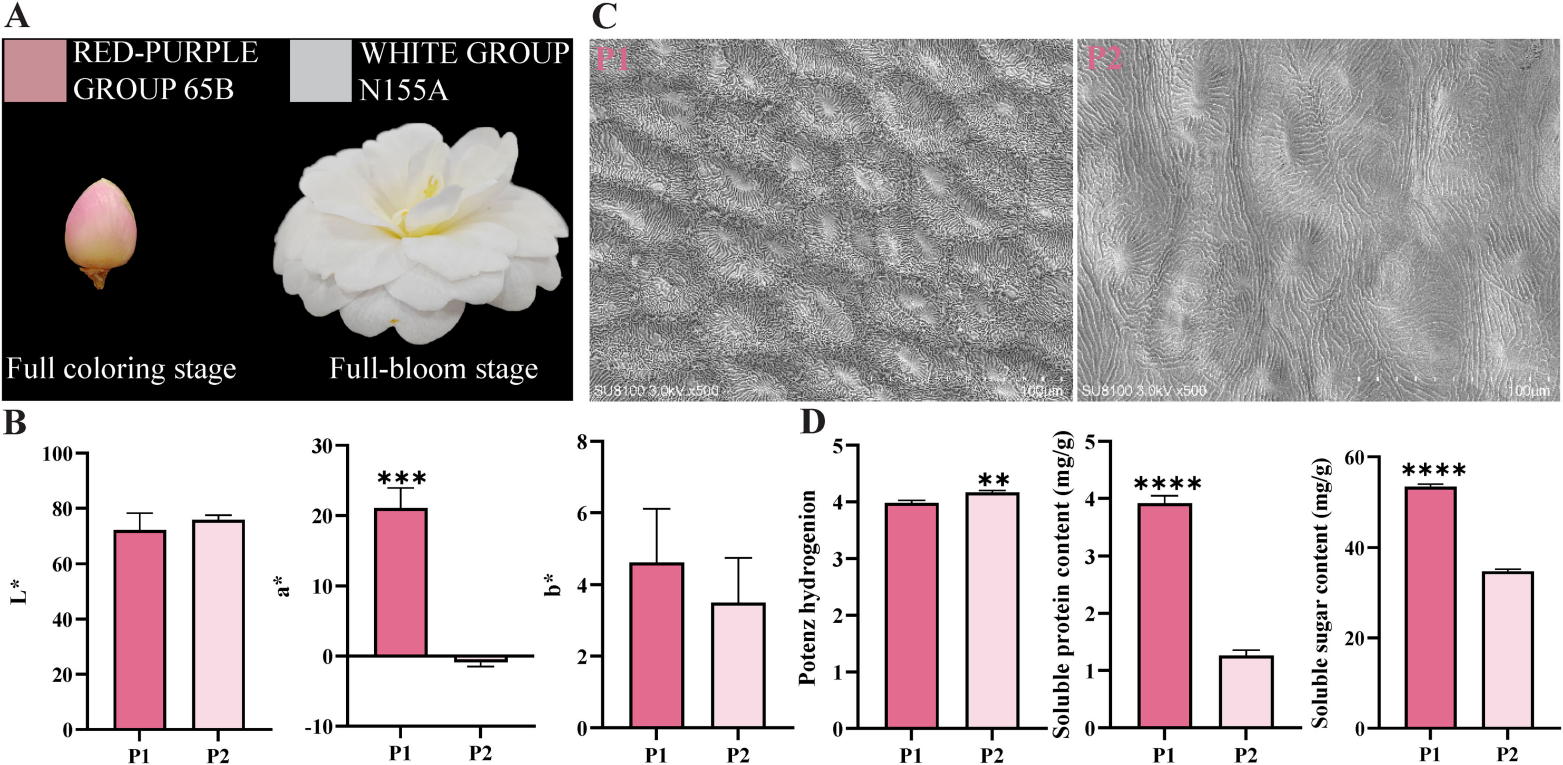
Phenotypic traits and indicators of the cellular physiological environment of ‘Tongzimian’ petals. **(A)** ‘Tongzimian’ petal phenotypes. **(B)** CIELAB color measurements of petals at two developmental stages. **(C)** Scanning electron microscopy images of the upper epidermis of ‘Tongzimian’ petals. **(D)** Indicators of the intracellular physiological environment of ‘Tongzimian’ petals. Statistical significance was determined using Student’s t-test (**P<0.01, ***P<0.001,****P<0.0001). P1: full coloring stage; P2: full-bloom stage.

During the flowering process of ‘Tongzimian’, its petals transition from the RED-PURPLE GROUP 65B at P1 to the WHITE GROUP N155A at P2 (**Figure 1A**). Correspondingly, the a* (red-greenness) values were positive at P1 but became negative at P2, indicating a significant color change between the two stages (**Figure 1B**). Scanning electron microscopy revealed that the upper epidermal cells of ‘Tongzimian’ petals are slightly concave, oval-shaped, and decorated with striped ornamentation (**Figure 1C**). Notably, the upper epidermal cells at P2 were nearly twice the size of those at P1, while the folding degree was most prominent at P1, suggesting structural changes accompany color fading. Since the cellular physiological environment influences flower color expression, we measured three key physiological indicators: pH, soluble sugar, and soluble protein. Both the P1 and P2 stages showed a pH of approximately 4, with P2 markedly higher than P1 (**Figure 1D**). The contents of soluble sugar and soluble protein were significantly higher at P1 than at P2, indicating a potential relationship between decreasing soluble matter and petal color fading.

### 3.2 Cyanidin is the main anthocyanin in the petals of ‘Tongzimian’

**Figure 2:**
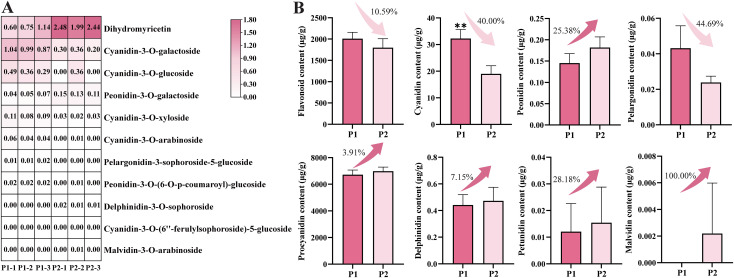
Identification of anthocyanins in ‘Tongzimian’. **(A)** Heatmap of anthocyanins in ‘Tongzimian’. **(B)** The content of flavonoid, cyanidin, peonidin, delphinidin, pelargonidin, procyanidin, petunidin, and malvidin in the flowers of ‘Tongzimian’. Statistical significance was determined using Student’s t-test (**P<0.01). P1: full coloring stage; P2: full bloom stage; -1, -2, -3 indicate biological replicates.

We employed LC-MS/MS to quantify anthocyanins in the petals of ‘Tongzimian’ at different developmental stages. Results showed that 41 anthocyanin metabolites were detected in the petals of ‘Tongzimian’, including 12 cyanidins, 8 flavonoids, 7 delphinidins, 6 procyanidins, 3 pelargonidins, 3 peonidins, 1 petunidin, and 1 malvidin. Among these, 11 differential anthocyanin metabolites were identified between the two developmental stages, with cyanidins accounting for the majority. Notably, the concentration of all differential metabolites was less than 5 μg/g (**Figure 2A**). Secondary classification analysis revealed that procyanidin was the most abundant anthocyanin-related metabolite in ‘Tongzimian’ petals, followed by flavonoids and cyanidin; cyanidin content showed a significant difference between the two periods (**Figure 2B**). This difference in cyanidin content is the key factor causing the fading of floral color in ‘Tongzimian’. Although procyanidins are colorless, they represent the most abundant class of anthocyanin-related metabolites in ‘Tongzimian’ petals and play an indirect role in color fading by competing with cyanidin for a common precursor.

### 3.3 Anthocyanidin synthase was weakly expressed in ‘Tongzimian’ at the bloom stage

**Figure 3:**
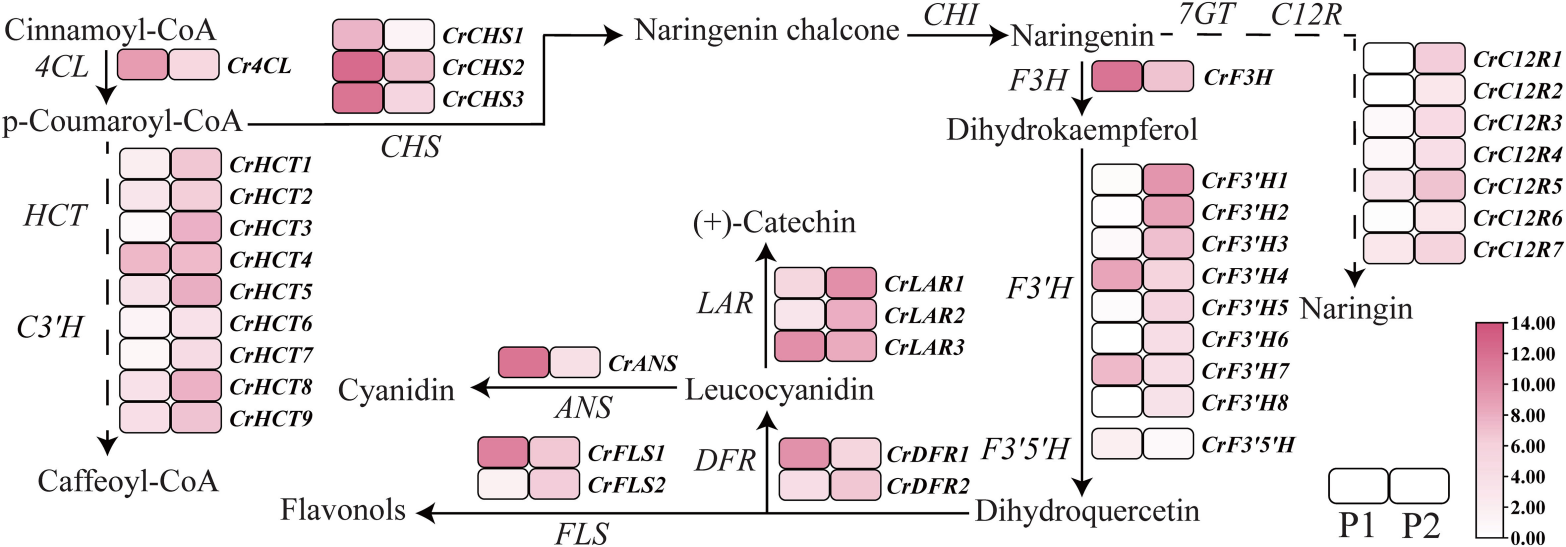
Overview of key structural genes in the anthocyanin biosynthesis pathway in ‘Tongzimian’. *4CL*, trans-cinnamate 4-monooxygenase; *HCT*, shikimate O-hydroxycinnamoyl transferase; *C3’H*, 5-O-(4-coumaroyl)-D-quinate 3’-monooxygenase; *CHS*, chalcone synthase; *CHI*, chalcone isomerase; *7GT*, flavanone 7-O-beta-glucosyltransferase; *C12R*, flavanone 7-O-glucoside 2’’-O-beta-L-rhamnosyltransferase; *F3H*, naringenin 3-dioxygenase; *F3’H*, flavonoid 3’-monooxygenase; *F3’5’H*, flavonoid 3’,5’-hydroxylase; *DFR*, bifunctional dihydroflavonol 4-reductase; *FLS*, flavonol synthase; *ANS*, anthocyanidin synthase; *LAR*, leucoanthocyanidin reductase. P1: full coloring stage; P2: full-bloom stage.

To further clarify the molecular mechanism behind these metabolite changes, we sequenced the petal transcriptome at two developmental stages. As a result, a total of 40.74 GB of clean data was generated, with 92.76%–93.32% of bases scoring Q30. In the petals of ‘Tongzimian’, 21,106 transcripts were identified, among which 6,495 were found to be differentially expressed. As anthocyanins are primary contributors to *Camellia*’s red color, we focused our analysis on structural genes in the anthocyanin biosynthesis pathway. Through this approach, 38 relevant structural genes were detected, including 1 *4CL*, 3 *CHSs*, 9 *HCTs*, 8 *F3’Hs*, 1 *F3’5’H*, 1 *F3H*, 2 *DFRs*, 2 *FLSs*, 3 *LARs*, 1 *ANS*, and 7 *C12Rs* (**Figure 3**). Comparing metabolite content, cyanidin levels at P2 were significantly lower than at P1, in ‘Tongzimian’, while procyanidin content was slightly higher. Because procyanidin and cyanidin share a common biosynthetic pathway up to leucocyanidin—conversion to procyanidin by *LAR* or to cyanidin by *ANS*. Therefore, increased LAR expression promotes procyanidin synthesis over cyanidin, whereas higher *ANS* expression favors cyanidin production. Supporting this, the expression level of *CrANS* was significantly lower at P2, while those of *CrLAR1* and *CrLAR2* increased. These findings clarify that reduced *CrANS* and increased *CrLAR1*/*CrLAR2* expression redirect metabolic flux from cyanidin, causing reduced red pigmentation and color fading in ‘Tongzimian’, with *CrANS* downregulation being a central causal factor.

**Figure 4:**
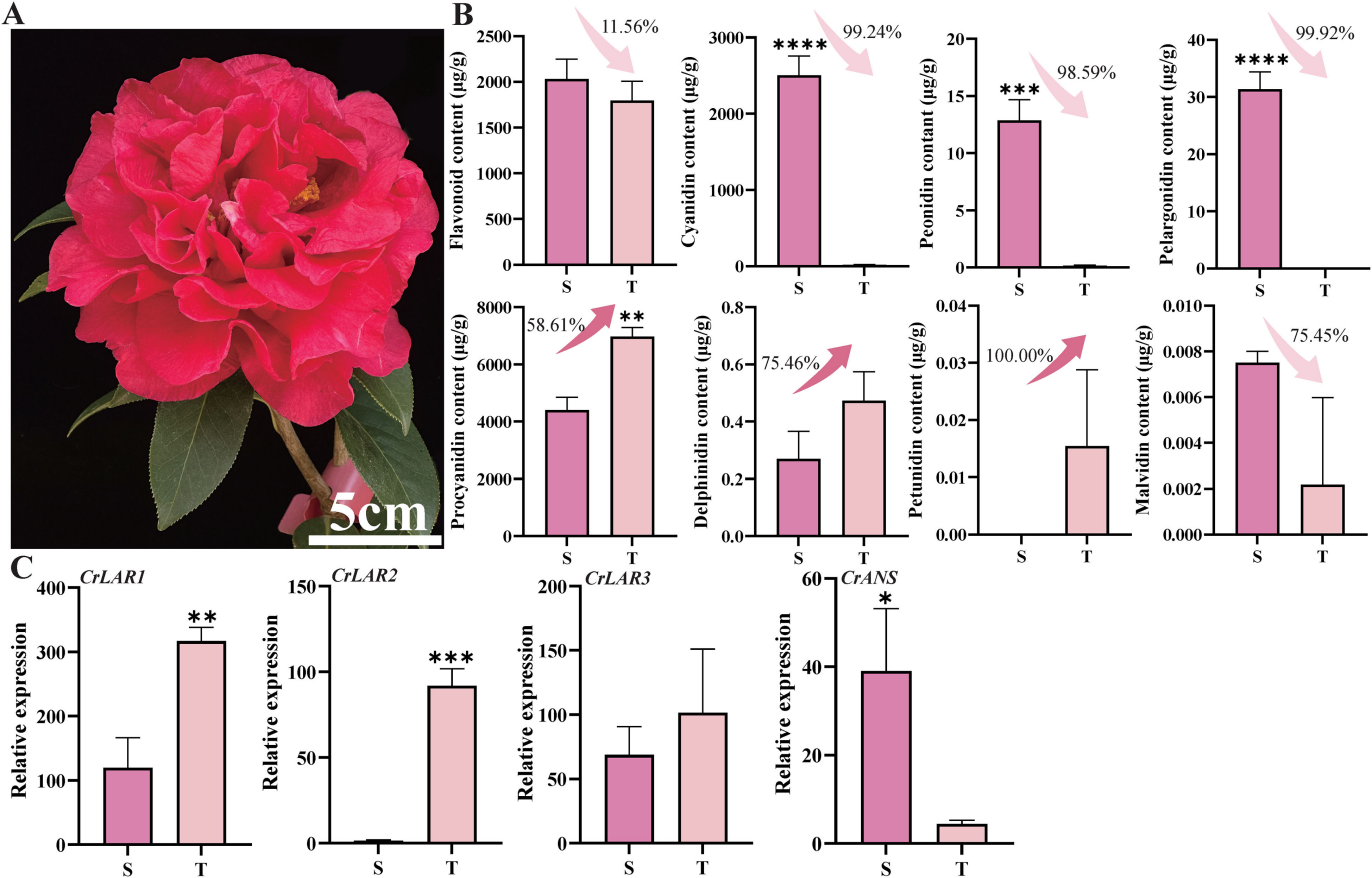
‘Tongzimian’ and ‘Shizitou’ multi-omics analysis. **(A)** ‘Shizitou’ petal phenotypes. **(B)**. **(B)** The content of flavonoid, cyanidin, peonidin, delphinidin, pelargonidin, procyanidin, petunidin, and malvidin in flowers of ‘Shizitou’ and ‘Tongzimian’. **(C)**. *CrLAR1*, *CrLAR2*, *CrLAR3*, and *CrANS* expression pattern analysis. S: ‘Shizitou’; T: ‘Tongzimian’; Statistical significance was determined using Student’s t-test (*P<0.05,**P<0.01, ***P<0.001, ****P<0.0001).

To provide comparative support for these findings, we conducted an omics analysis using pure red ‘Shizitou’ as a control (**Figure 4A**). The content of cyanidin, peonidin, and pelargonidin in ‘Shizitou’ was significantly higher than in ‘Tongzimian’, with cyanidin showing the greatest difference (**Figure 4B**). Procyanidin content was lower in ‘Shizitou’ than in ‘Tongzimian’. Transcriptome analysis shows *CrLAR1* and *CrLAR2* expression are significantly higher, and *CrANS* expression is markedly lower in ‘Tongzimian’ than in ‘Shizitou’ (**Figure 4C**). These findings confirm that reduced *CrANS* expression increases flux toward procyanidins, leading to lighter flower coloration in ‘Tongzimian’.

### 3.4 Phylogenetic analysis of CrANS

**Figure 5:**
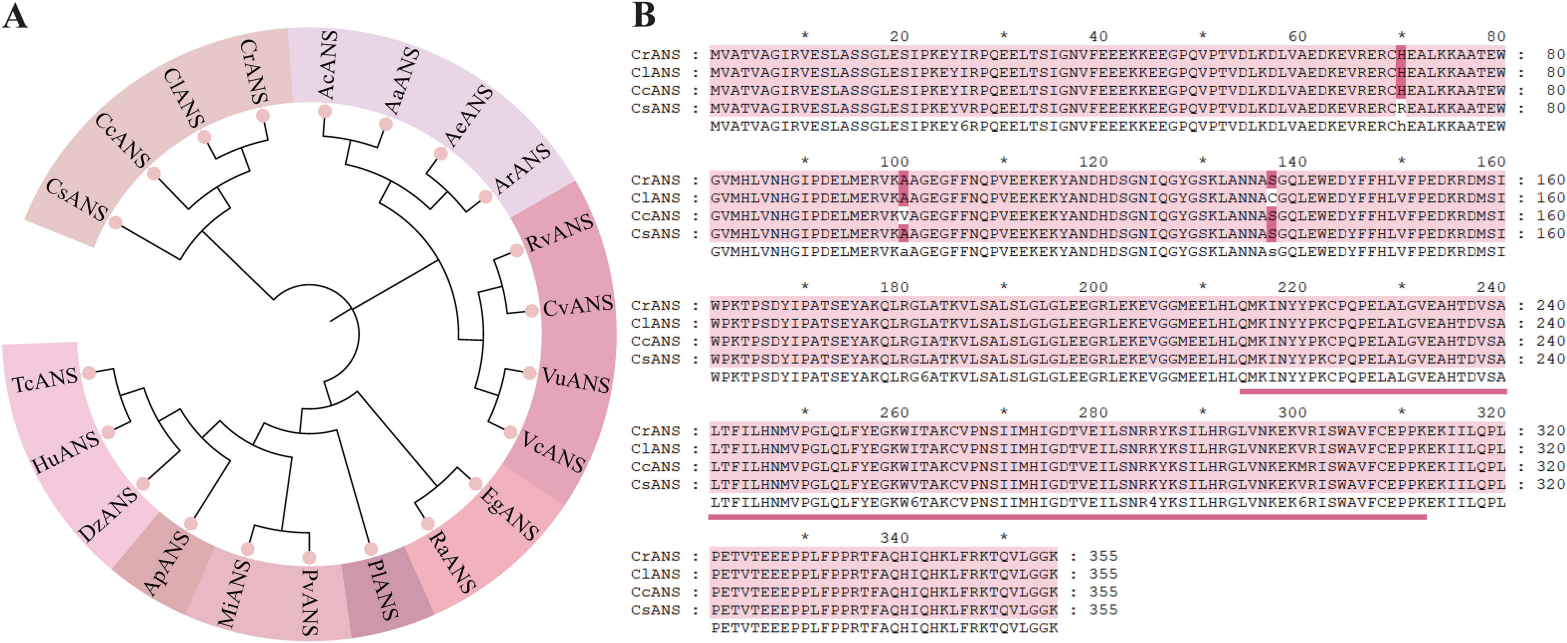
**(A)** Phylogenetic analysis of CrANS proteins from different species. GenBank accession numbers are as follows: CsANS (*Camellia sinensis* QIM55351.1), CcANS (*Camellia chekiangoleosa* AFC37246.1), ClANS (*Camellia lanceoleosa* KAI7988436.1), AcANS (*Actinidia chinensis* AGV53047.1), AaANS (*Actinidia arguta* QGN03641.1), AeANS (*Actinidia eriantha* XP_057486995.1), ArANS (*Actinidia rufa* GFZ02163.1), RVANS (*Rhododendron vialii* XP_058221777.1), CvANS (*Calluna vulgaris* CAM5866044.1), VuANS (*Vaccinium uliginosum* UAJ71238.1), VcANS (*Vaccinium corymbosum* AYC35393.1), EgANS *(Eucalyptus grandis* XP_010053365.2), RaANS (*Rhodamnia argentea* XP_030524727.1), PlANS (*Paeonia lactiflora* QIC54082.1), PvANS (*Pistacia vera* XP_031284664.1), MiANS (*Mangifera indica* XP_044486033.1), ApANS (*Acer palmatum* AWN08246.1), DzANS (*Durio zibethinus* XP_022736758.1), HuANS (*Herrania umbratica* XP_021279732.1), TcANS (*Theobroma cacao* EOY24568.1). **(B)** Amino acid sequence alignment of the CrANS protein in *C. reticulata* ‘Tongzimian’ with proteins from other species.

Sequencing analysis revealed that *CrANS1* contains a 1068-nucleotide open reading frame (ORF) and encodes 355 amino acids. As there are currently no studies on the function of ANS in *C. reticulata*, we conducted a BLAST search against the NCBI database to identify ANS homologs from other species. The sequences of these identified homologs were downloaded from the database, and a phylogenetic analysis was performed, including CrANS. Phylogenetic results demonstrated that the CrANS protein clustered into a single clade with CsANS, CcANS, and ClANS proteins (**Figure 5A**), indicating a close evolutionary relationship between CrANS and ANS proteins from other *Camellia* species. Additionally, sequence alignment analysis revealed that CrANS shares over 95% amino acid sequence identity with ClANS, CcANS, and CsANS (**Figure 5B**), suggesting that the ANS gene is highly conserved within the *Camellia* genus.

### 3.5 Functional analysis of *CrANS*

**Figure 6:**
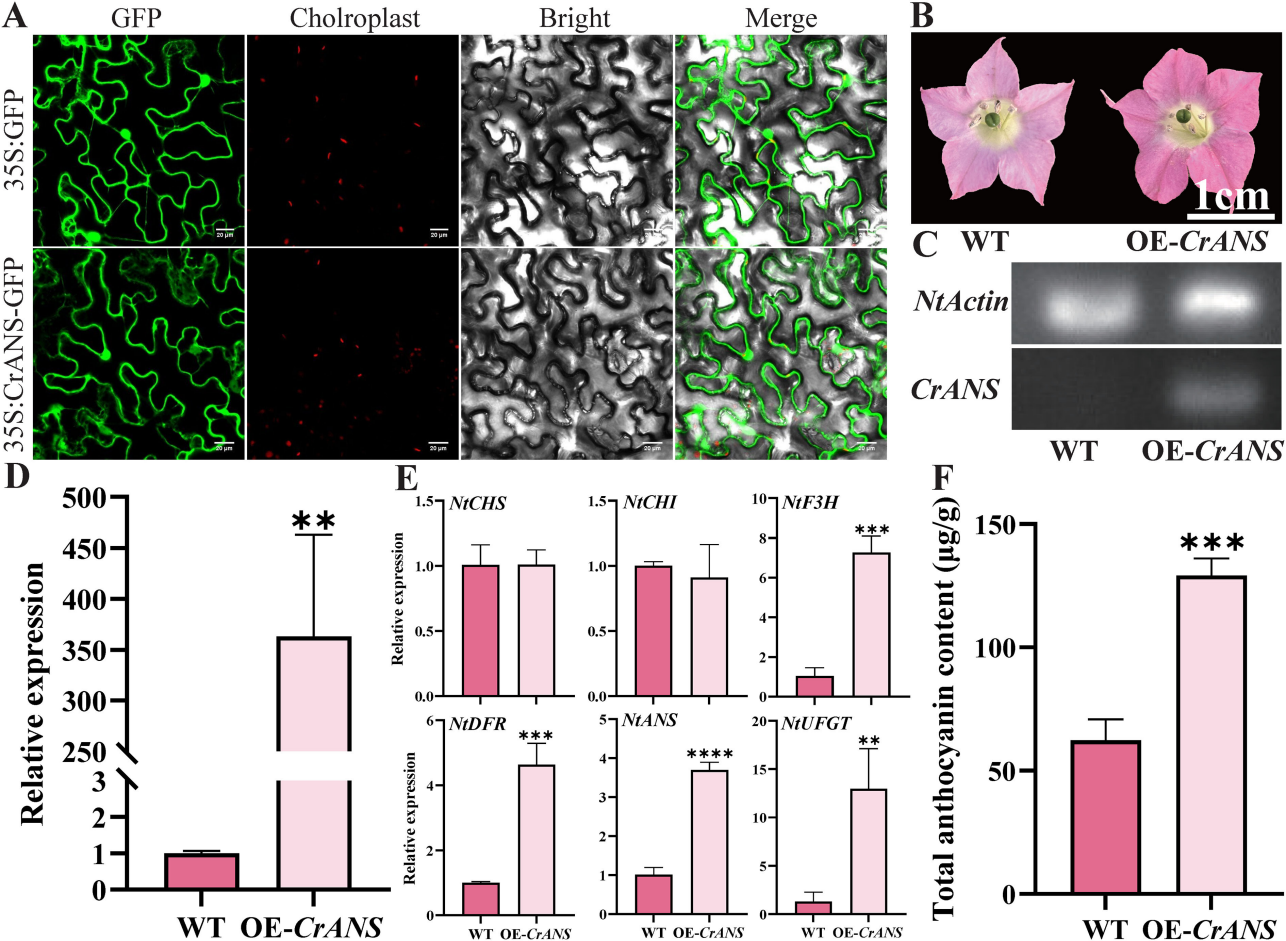
Effects of overexpression of *CrANS* in transgenic tobacco. **(A)** Subcellular localization of GFP fusion protein in tobacco leaves. Scale bar, 20 μm. **(B)** The Phenotype of flowers of wild-type **(WT)** and *CrANS* transgenic tobacco flowers. **(C)** Analysis of the expression of *CrANS* in WT and *CrANS* transgenic tobacco flowers. *NtActin* was used as a housekeeping gene. **(D)**. The relative expression of *CrANS* in WT and *CrANS* transgenic tobacco flowers. **(E)** Expression patterns of 6 structural genes in flowers of WT and *CrANS* transgenic tobacco. **(F)** Determination of the anthocyanin contents. Error bars indicate ± SD from three repeats. Statistical significance was determined using Student’s t-test (**P<0.01, ***P<0.001, ****P<0.0001).

To determine CrANS’s subcellular localization, we constructed a 35S: CrANS-GFP vector and transiently introduced it into tobacco leaf epidermal cells, with an empty 35S: GFP vector as a control. In controls, green fluorescence appeared throughout the cell, whereas for 35S: CrANS-GFP, fluorescence was restricted to the nucleus and cytoplasm. This indicates CrANS localizes to the nucleus and cytoplasm (**Figure 6A**).

Following the subcellular localization study, we assessed the functional role of *CrANS* by introducing the 35S promoter-*CrANS* module into tobacco via *Agrobacterium-*mediated transformation. This generated transgenic lines. The redder appearance of transgenic corolla tubes compared to WT flowers indicated an effect of *CrANS* on flower pigmentation (**Figure 6B**). RT-PCR and qRT-PCR showed *CrANS* was barely expressed in WT petals (**Figures 6C, D**). Additionally, anthocyanin biosynthesis-related genes *NtF3H*, *NtDFR*, *NtANS*, and *NtUFGT* were significantly upregulated in *CrANS*-overexpressing flowers (**Figure 6E**). Anthocyanin content in corollas was significantly higher in *CrANS*-overexpressing plants than in WT (**Figure 6F**), supporting a role for *CrANS* in regulating anthocyanin accumulation.

## 4 Discussion

This study elucidates the molecular basis of flower color fading in C. reticulata ‘Tongzimian’. Specifically, downregulation of the key structural gene, *CrANS*, is identified as the primary cause. This diminished *CrANS* expression inhibits anthocyanin biosynthesis, significantly reducing petal anthocyanin content and redirecting flavonoid precursors toward procyanidin synthesis rather than the anthocyanin pathway. Concurrently, shifts in the cellular physiological milieu synergistically accelerate fading. These findings define the biochemical and genetic determinants of the ‘Tongzimian’ phenotype, clarify the metabolic reallocation between compounds, and offer theoretical guidance and molecular targets for Theaceae flower-color breeding.

In this study, RHCSS was used to assess ‘Tongzimian’ petals across two developmental stages. The results showed that flower color shifted from the red purplish group at P1 to the white group at P2 (**Figure 1A**). This transition was supported by analysis of flower color parameters, which revealed a significant decline in the a* value during flowering (**Figure 1B**). Taken together, these observations, led us to hypothesize that the lightening of ‘Tongzimian’ flower color is due to a decrease in the pigments responsible for the red coloration. Supporting this, anthocyanin-targeted metabolomics results showed a significant reduction in cyanidin content (**Figures 2A, B**), a pigment responsible for conferring red coloration to petals. This finding aligns with the pattern observed in *Lycoris longituba* petals, where cyanidin content gradually decreases during development, leading to petal fading (Feng et al., 2024). Interestingly, despite the reduction in cyanidin content, levels of procyanidin — downstream products of the anthocyanin biosynthetic pathway — did not decrease in tandem. Therefore, the fading of ‘Tongzimian’ flower color may not result from comprehensive inhibition of the upstream anthocyanin biosynthetic pathway. Instead, it may reflect changes in the expression or enzyme activity of the downstream genes *ANS* and *UFGT*, which specifically catalyze the final steps of anthocyanin biosynthesis.

**Figure 7:**
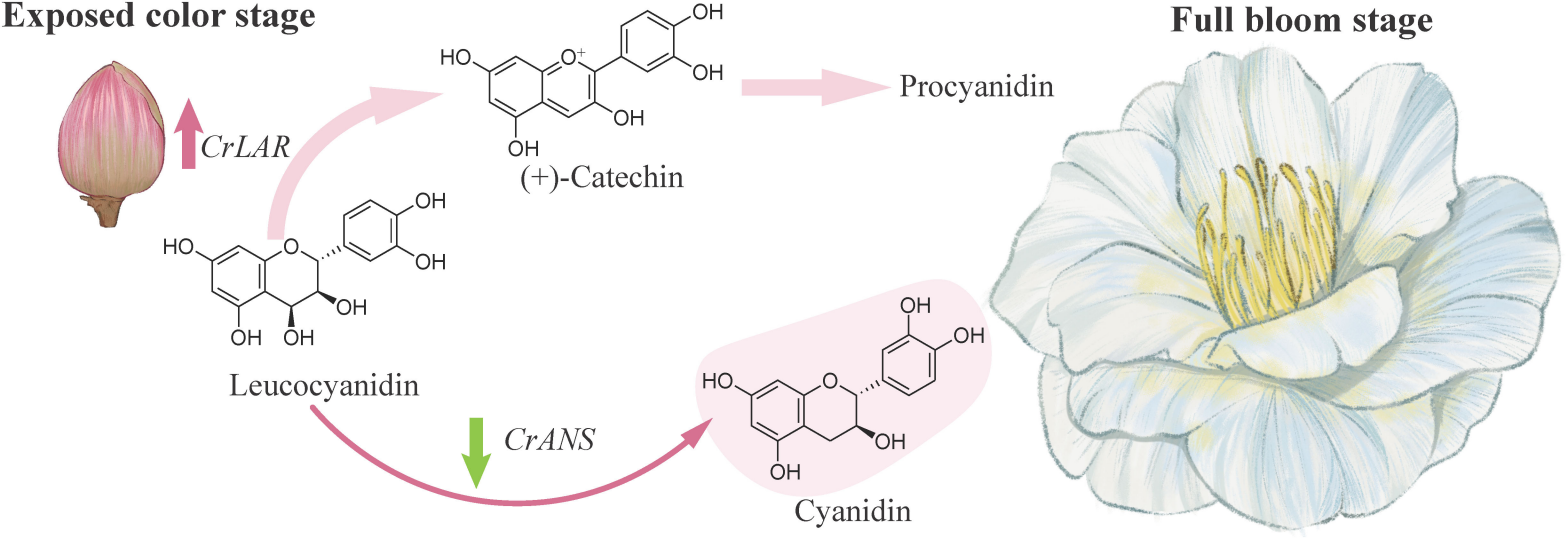
Effects of overexpression of *CrANS* in transgenic tobacco.

Comparative transcriptome analysis of ‘Tongzimian’ petals at stages P1 and P2 revealed 6,495 DEGs, including 38 structural genes in the anthocyanin biosynthesis pathway. Integrating these transcriptomic data with anthocyanin-targeted metabolomics, we identified key structural genes that specifically catalyze the biosynthesis of cyanidin and procyanidin. Notably, both Anthocyanidin Synthase (ANS) and Leucoanthocyanidin Reductase (LAR) utilize leucocyanidin as a substrate, yielding colored anthocyanins and colorless procyanidin, respectively (Cheng et al., 2021; Xu et al., 2021; Yang et al., 2022). Furthermore, our analysis showed that *CrANS*, which catalyzes the production of colored cyanidin, was downregulated at stage P2, while *CrLAR1* and *CrLAR2*, which convert leucocyanidin to colorless procyanidin for procyanidin biosynthesis, were upregulated. This pattern suggests a diversion of leucocyanidin towards the colorless pathway during late flowering. Based on these findings, we hypothesize that reduced *CrANS* expression diminishes colored cyanidin synthesis, whereas increased *CrLAR* expression channels more leucocyanidin into procyanidins, collectively contributing to the paler flower color (**Figure 7**). In addition, comparison with the darker ‘Shizitou’ variety further indicates that lower *CrANS* expression directly causes the color lightening observed in ‘Tongzimian’.

Functional analysis of the key gene *CrANS* during the color variation process in the ‘Tongzimian’. *ANS* genes are widely distributed among higher plants, and their amino acid sequences show a high degree of homology. Protein sequence alignment confirmed high homology (>95%) between CrANS and functionally characterized ANS enzymes from other *Camellia* species (e.g., *C. lanceoleosa*, *C. chekiangoleosa*, *C. sinensis*), underscoring its conserved catalytic role (**Figures 5A, B**). To preliminarily validate the *CrANS* function, an overexpression experiment was conducted in tobacco. The results showed that overexpression of *CrANS* upregulates downstream anthocyanin biosynthesis genes, thereby significantly increasing anthocyanin accumulation and leading to deeper coloration of the tobacco corolla. These results provide preliminary evidence that *CrANS* promotes anthocyanin synthesis. However, relying solely on heterologous overexpression in tobacco limits understanding of *CrANS’s* endogenous regulatory mechanisms within *Camellia* species. Further in-depth analyses are needed to elucidate its precise role in the native context of *Camellia* flower color regulation.

In addition, the shape of the epidermal cells of the petals and the cellular physiological environment play an important role in determining the color of the flower (Wang et al., 2023; Yuan et al., 2023). In this study, both developmental periods of ‘Tongzimian’ petals had oval cells, concave inward with folds, but P1 petals had significantly more folds than P2. This greater folding allows more light to enter the cells, resulting in a deeper petal color (Chen et al., 2020). Thus, the darker petal color during the P1 in ‘Tongzimian’ may be due to this increase in folds. Furthermore, pH value, soluble sugars, and soluble proteins were higher in P1 petals than in P2. These increased contents of soluble sugars and soluble proteins may help maintain or enhance the flower color of ‘Tongzimian’, as similarly observed in *Malus pumila* (Wang et al., 2021) and *Vaccinium uliginosum* (Zeng et al., 2023) species. In summary, it is hypothesized that during flowering, decreases in soluble protein and soluble sugar may raise pH and reduce anthocyanin synthesis; combined with larger epidermal cells and fewer folds in the petals, these changes contribute to the fading of flower color. Although other genes and physiological factors (e.g., pH, cell shape) also contribute, *CrANS* represents a critical regulatory point in cyanidin reduction.

## 5 Conclusion

The color of ‘Tongzimian’ petals transitions from pink to white during flowering. During this process, the epidermal cells continuously enlarge, with reduced folds; the pH value increases; and the levels of soluble sugars and soluble proteins decrease. Procyanidins serve as the main pigment components in its petals, while cyanidin acts as the key substance driving floral color fading. The *CrANS* gene has been successfully cloned and shows significantly higher expression at the fully colored stage than at the full-bloom stage. Furthermore, overexpression of *CrANS* enhances anthocyanin accumulation in tobacco corollas, resulting in darker flower color. Collectively, these findings provide a theoretical basis for breeding new *C. reticulata* cultivars.

## Data Availability

The original contributions presented in the study are included in the article/**Supplementary Material**. Further inquiries can be directed to the corresponding author/s.
